# Lymphovenous Bypass: Past, Present, and Future

**DOI:** 10.1055/a-2897-0429

**Published:** 2026-08-21

**Authors:** Erin Brown, Jin Geun Kwon, Changsik John Pak, Joon Pio Hong

**Affiliations:** 1 Division of Plastic and Reconstructive SurgeryUniversity of British ColumbiaVancouverBritish ColumbiaCanada; 2 Department of Plastic SurgeryAsan Medical Center, University of Ulsan College of MedicineUlsanUlsanKorea (the Republic of)

**Keywords:** reconstruction/lymphedema, lymphedema–lymphovenous shunting, lymphedema–lymphatic physiology, lymphedema, lymphovenous bypass, immediate lymphatic reconstruction, lymphedema surgery, lymphangiogenesis, lymphatic imaging

## Abstract

Lymphovenous bypass (LVB) has evolved from an experimental concept to a widely adopted microsurgical intervention for secondary lymphedema. Advances in imaging, surgical technique, and understanding of lymphatic anatomy have expanded its indications, yet its long-term efficacy and ideal application remain incompletely defined. This review examines the historical development, anatomic foundations, and physiologic principles underlying lymphovenous anastomosis. Key topics include the influence of patient-specific lymphatic anatomy and lymphangiogenesis on surgical outcomes, the rheological determinants of lymphatic–venous flow, and evolving techniques such as immediate lymphatic reconstruction (ILR). Both objective and patient-reported outcome (PROM) data are critically appraised, and persisting gaps in knowledge are identified. Emerging evidence demonstrates that lymphangiogenesis and collateral formation after lymphatic injury are highly variable and strongly influence both the development of lymphedema and the success of LVB. Rheological analysis highlights the exponential impact of vessel diameter and flow resistance on anastomotic performance. While LVB yields modest objective limb volume reductions (6–33%), consistent improvements in quality of life and symptom burden are observed. Evidence supporting ILR suggests short-term benefit in reducing lymphedema incidence, though longer-term follow-up indicates attenuation of this effect. LVB represents a mature and evidence-supported component of reconstructive microsurgery, yet substantial heterogeneity persists in patient selection, technical execution, and outcome evaluation. Future progress will depend on integrating precision imaging, standardized outcome measures, and deeper understanding of lymphatic biology to enable personalized, anatomy-driven interventions that restore physiologic lymphatic function.

## Introduction

The basic pathophysiology and typical progression of lymphedema as a chronic, progressive disorder characterized by the accumulation of interstitial lymphatic fluid and secondary tissue fibrosis is well-established. Traditional management has relied heavily on conservative therapies such as compression garments, manual lymphatic drainage, and decongestive physiotherapy. Our growing understanding of the anatomy and physiology of the lymphatic system provides the theoretical opportunity to intervene in the management of both congenital and secondary lymphedema. Particularly for secondary lymphedema, resulting from surgical disruption of the normal lymphatic pathways, the potential of bypassing the disruption by returning the lymphatic fluid to the venous system prior to the typical connection of the thoracic duct to the vena cava can reduce the accumulation of excess interstitial fluid, and the secondary sequela of lymphedema is theoretically straightforward. This conceptually simple intervention has been challenging to achieve despite a greater than 150-year history of the understanding of the anatomy of the lymphatic system. 1 This review will detail the history of lymphovenous bypass (LVB), the introduction of supermicrosurgery, and the challenges remaining to realize the goal of “cure” of lymphedema.

Over the past five decades, the field of lymphatic surgery has undergone a transformative evolution. What began as speculative efforts to bypass obstructed lymphatics using crude shunts and macrosurgical techniques has matured into a sophisticated subspecialty of supermicrosurgical reconstruction, guided by high-resolution imaging and detailed anatomical mapping. Among the most significant innovations is LVB—also termed lymphovenous anastomosis (LVA)—a physiologic procedure that reroutes lymphatic flow directly into the venous system via microsurgical connections between lymphatic collectors and small venules. First attempted in the 1960s, LVB now offers measurable benefit in appropriately selected patients, particularly in early-stage or functionally preserved lymphatic disease.

The modern success of LVB has been enabled by parallel advances in lymphatic imaging, particularly indocyanine green (ICG) lymphography, and by the introduction of the lymphosome concept, which defines the body's superficial lymphatic territories in a predictable, topographically consistent fashion. Together, these tools have shifted lymphatic surgery from empiricism to an anatomically targeted, physiologically informed intervention. However, the most efficacious method of redirecting lymphatic fluid to the venous system remains unclear. Moreover, there remain imprecise metrics for determining the outcomes following this surgery.

Due to the stark pathophysiological differences between congenital and acquired lymphedema, this review will focus on the role of LVB in secondary lymphedema resulting from “traumatic” lymphatic disruption, such as lymphadenectomy and radiotherapy. Although some cases of congenital lymphedema may benefit from bypass surgery, the broader dysfunction of the lymphatics with a congenital or developmental etiology may preclude the effective use of bypass to consistently address this form of lymphedema. Similarly, lymphedema resulting from filariasis infection is also not likely to be appropriate for management with bypass surgery due to the more diffuse lymphatic disruption.

## Historical Understanding of the Lymphatic System Anatomy

Although evidence suggests that intra-abdominal chyle, or “white blood,” was described as early as the 5th century BC by Hippocrates, the foundational anatomical descriptions of the lymphatic system of the extremities emerged in the 17th century through the observations of Thomas Bartholin and Olaus Rudbeck. Subsequent anatomical specimens prepared by Frederik Ruysch in the late 17th and early 18th centuries using liquor balsamicum further elucidated lymphatic anatomy, including the presence of intraluminal valves. In the late 18th century, Paolo Mascagni provided a comprehensive description of the superficial and deep lymphatic systems and is widely credited with producing the first detailed anatomical characterization of the lymphatic system. In the 19th century, Marie Philibert Constant Sappey further advanced the field through his descriptions of lymphatic drainage territories and nodal basins, concepts that underpin the modern understanding of lymphosomes. 1

## The Lymphosome Concept: Anatomical Foundations and Surgical Relevance to Lymphovenous Anastomosis

The basic conceptual principles of LVB, returning lymphatic fluid to the circulatory system distal to a zone of obstruction, are remarkably intuitive, but the detailed anatomic understanding required to begin actualizing this goal was elusive. Among the most influential developments in our understanding of the functional anatomy of the lymphatic system is the delineation of lymphosomes—discrete anatomical territories of lymphatic drainage that correspond to specific regional lymph node basins. This concept, first systematically characterized by Suami and colleagues, provides a detailed functional map of lymphatic flow, analogous to the angiosome model in plastic surgery. 2 3

Using cadaveric microinjection techniques with radiopaque contrast agents followed by high-resolution micro-CT imaging, Suami et al demonstrated that the skin and subcutaneous tissues of the human body are organized into reproducible lymphatic territories. These lymphosomes drain via distinct superficial collecting vessels to predictable nodal groups (e.g., axillary, inguinal, cervical). Importantly, these territories are largely non-overlapping under physiologic conditions, with crossover or collateral flow occurring primarily in response to obstruction, trauma, or surgical disruption. 2 This pattern of anatomical specificity and the resultant dysfunction that arises from injury is similar to cortical cerebrovascular accidents. Like the cortical plasticity observed in clinical recovery in the brain, the ability of the lymphatic system to utilize crossover and collateral pathways can reduce the impact of disruptions of specific lymphosomal drainage. This ability to realize clinical recovery is an important consideration in understanding the natural history of secondary lymphedema—recovery is possible—and to better conceptualize the timing and nature of lymphatic reconstruction.

The lymphosome model has since been validated through multiple clinical modalities, including ICG fluorescence lymphography, lymphoscintigraphy, and intraoperative mapping during oncologic and reconstructive procedures. 4 5 6 The predictable architecture of lymphatic flow has proven instrumental in the evolution of LVB, providing both a diagnostic framework and a surgical roadmap. Moreover, it should be readily apparent that the principle of sentinel lymph node biopsy utilized in oncologic diagnostic procedures relies on the anatomic specificity of lymphatic drainage delineated in the lymphosome concept.

In the context of LVB, the lymphosome concept offers several theoretical and practical advantages:

- **Anatomical targeting:** By identifying the specific lymphosome affected by lymphatic compromise (e.g., postaxillary dissection upper limb lymphedema), surgeons can direct their dissection and imaging toward regions with the highest probability of locating functional collecting vessels.
- **Mapping rerouted drainage:** In cases of secondary lymphedema, lymphatic rerouting often follows adjacent lymphosome boundaries. Understanding this compensatory flow is critical for selecting optimal anastomosis sites and anticipating outcomes. 4
- **Surgical planning and staging:** In complex or high-stage disease, lymphosome mapping supports the potential strategic placement of multiple LVBs or the combination of LVB with vascularized lymph node transfer, allowing individualized and anatomically sound treatment plans.
- **Assessment of postoperative function:** Postintervention ICG lymphography can be more precisely interpreted using the lymphosome framework, with restoration of linear flow within previously affected territories serving as an objective marker of surgical success.

The incorporation of the lymphosome concept into the current understanding of lymphatic function and dysfunction represents a pivotal step in the evolution of empirically guided lymphedema management to one grounded in region-specific anatomic interventions. As techniques and imaging modalities continue to evolve, lymphosome-based mapping is likely to remain integral to the standardization, teaching, and optimization of lymphatic microsurgery.

## Historical Development of Lymphovenous Bypass Surgery

As noted previously, the concept of redirecting lymphatic flow into the venous system dates back nearly a century, but only in recent decades has LVB become a realistic microsurgical treatment for lymphedema. Its development has been driven by advances in microsurgery, imaging, and the aforementioned anatomical understanding of the lymphatic system.

## Early Experimental Foundations (1930s–1960s)

The earliest investigations into lymphovenous communications were largely experimental. In the 1930s, Jacobson and Suarez introduced the concept of microvascular anastomosis in animals. 7 The first attempts to anastomose lymphatic vessels to veins were performed in the 1940s and 1950s in canine models, demonstrating that lymphatic–venous connections could alleviate lymphatic obstruction. In 1963, Nielubowicz and Olszewski performed the first lymphovenous shunt in humans, implanting a silicone tube between a lymphatic vessel and the venous system in patients with lower limb lymphedema. 8 However, these early procedures were hampered by limited visualization of lymphatics, large vessel calibers, and high-pressure venous reflux, which led to poor patency and inconsistent clinical results.

## Emergence of Microsurgery (1970s–1980s)

The introduction of advanced microsurgical techniques in the 1970s enabled anastomosis of vessels less than 1 mm in diameter, which revolutionized the field. In 1977, O'Brien et al reported the first successful microsurgical lymphatic–venous anastomosis using an end-to-end technique in patients with secondary lymphedema after mastectomy. 9 This marked the beginning of a new era in physiologic lymphedema surgery.

During this period, techniques such as end-to-end and end-to-side LVBs were developed, but long-term results remained inconsistent due to challenges in identifying functional lymphatic vessels and preventing venous reflux. For example, Gloviczki and colleagues performed end-to-end LVBs in a canine model to determine the long-term patency over time. 10 Although they demonstrated technical proficiency in performing the 0.7- to 1.2-mm anastomoses, subsequent examination demonstrated that patency fell to 42% within 2 to 6 weeks. The inconsistent ability to achieve long-term patency led the authors to suggest “chances of success are better with several anastomoses performed in the early stages of lymphedema, before significant tissue fibrosis and complete loss of lymphatic valvular function develop.” Similarly, Puckett and colleagues reported 100% patency of LVB in a similar canine model at 7 days, but occlusion of all anastomoses by 21 days. 11 These animal studies that could directly examine long-term patency influenced the cohort of surgeons who advanced these techniques for subsequent clinical application.

## Technological Advancements and Refinement (1990s–2000s)

The 1990s and early 2000s saw limited widespread adoption of LVB, largely due to difficulties in intraoperative visualization of lymphatic vessels and selection of optimal anastomosis sites. However, interest resurged with the development of near-infrared fluorescence imaging and the clinical introduction of ICG lymphography. These advances allowed for real-time, high-resolution mapping of superficial lymphatic collectors and dermal backflow patterns.

Japanese surgeons, particularly Koshima et al 12 and Yamamoto et al, 4 were instrumental in refining supermicrosurgical LVB. Koshima's team popularized the use of ultrafine anastomoses between subdermal lymphatic collectors and venules as small as 0.3 mm in diameter, improving long-term patency and making the procedure feasible under local anesthesia. 13 The clinical experience and publications from these innovators provided the required “proof of principle”—LVA was a technically feasible intervention to realize the goal of redirecting lymphatic fluid to the venous system distal to the level of the obstruction to flow. Utilizing the understanding of the previously documented tendency for individual anastomoses to fail, there was a recommendation for multiple bypasses throughout the extremity. Although this was a dramatic step forward in the development of this treatment paradigm, it did not reliably respect the lymphosome anatomy as previously discussed, precluding an approach that selected the “most” appropriate lymphatics for rerouting. Another important consideration was the “method” of microvascular anastomosis. Although end-to-end anastomosis is the most common method of performing coaptations of arteries and veins in replantations and free tissue transfers, it was unclear if this approach was also ideal for LVA.

In addition to the critical role of innovators in driving the evolution of the field of LVB, the essential contribution of the specialized microsurgical equipment and operative microscopes should not be discounted.

Their role can be broken down into several key aspects:

- **Visualization** : Lymphatic vessels are typically 0.2 to 0.8 mm in diameter, often transparent, and difficult to distinguish from surrounding tissue. High-magnification operative microscopes (20–40 × ) allow the surgeon to identify these delicate structures and confirm patency when suturing walls only a few microns thick.
- **Instrumentation** : Although standard microsurgical instruments (e.g., fine jeweler's forceps, microscissors, vessel dilators) are designed to manipulate small and fragile arteries, veins, and nerves without crushing them, they are frequently inadequate for lymphatic surgery. Specifically, LVB requires the use of supermicrosurgical needle drivers, forceps, scissors, and microclamps for the utilization of 11–0 and 12–0 sutures.
- **Imaging and navigation support** : In addition to the ICG lymphography utilized to plan for LVB, the integration of this functionality into the microscope allows real-time visualization of functional lymphatic channels, as well as the assessment of patency following LVB.

In short, specialized microsurgical equipment and operative microscopes make LVB “possible.” They allow surgeons to reliably identify, handle, and anastomose fragile lymphatic and venous vessels at the submillimeter scale, directly impacting both the technical success of the surgery and patient outcomes.

## Methods of Lymphovenous Anastomosis

The principal technical forms of LVA are end-to-end, side-to-end, end-to-side, and side-to-side configurations. Additional technical refinements include sequential anastomosis (multiple lymphatics to a single venule), lambda-shaped anastomosis, and adjuncts such as the suture-stent technique and intraoperative distal compression to optimize patency and flow. 13 14 15 16 17 18 19 20

Comparative clinical studies indicate that side-to-end anastomosis may provide superior volume reduction compared with end-to-end, particularly in advanced-stage lymphedema, while both are effective in early-stage disease. The choice of configuration is often determined by the relative size and quality of the lymphatic and venous vessels, with side-to-end and end-to-side preferred when there is a significant size discrepancy. Experimental data suggest that lymphaticovenular anastomosis achieves higher postoperative patency than lymphaticovenous implantation. 14 15 19 20

Technical modifications such as the suture-stent technique and lambda-shaped anastomosis have been shown to improve intraoperative success and facilitate multiple bypasses, but there is insufficient evidence to declare any one adjunct as definitively superior in long-term clinical outcomes. 13 17 18

In summary, side-to-end and end-to-end are the most commonly used and studied LVB techniques, with side-to-end showing some advantage in advanced lymphedema, but the optimal technique should be individualized based on intraoperative vessel characteristics and surgeon expertise. 14 15 19 Ultimately, our understanding of the appropriateness or efficacy of these methods of achieving LVB is predicated on limited data that are strongly biased by the concepts and experience of individual surgeons, and not by well-designed investigations of the rheology, patency, and ease of performance of these techniques. Nevertheless, it does appear that a variety of methods of vessel coaptation can be demonstrated to provide demonstrable intraoperative patency.

## Contemporary Practice (2010s–Present)

Over the past decade, LVB has evolved into a minimally invasive, physiologic reconstructive technique with increasing international adoption. The procedure is now commonly used in early-stage lymphedema and as an adjunct to debulking or lymph node transfer in more advanced disease.

Modern LVB involves

- Preoperative ICG mapping to identify functional lymphatics
- Targeted multilevel anastomoses (often in multiple lymphosomes) *
- End-to-end or end-to-side anastomotic techniques using 11–0 or 12–0 nylon sutures
- Intraoperative assessment of flow using ICG or blue dye.

Clinical outcomes have improved with these refinements. Multiple prospective studies now support the use of LVB in reducing limb volume, improving quality of life, and reducing infection rates, especially when performed in early-stage disease. 21 22 23 Interestingly, a recent meta-analysis documented a stage-independent benefit of LVB for the management of both upper and lower extremity lymphedema. 24 Although not statistically significant, the data suggested a larger benefit for patients with more advanced disease, which is surprising given the potential absence of patent lymphatics for consideration of bypass in individuals with Stage III or fibrotic/non-pitting disease. Nevertheless, there is less consistent data regarding the impact of LVB in decreasing the size or volume of the treated limbs in some more recent studies. 25 26 27 Taken as a whole, there is compelling evidence to support the efficacy of LVB; however, the consistency of this effect varies. High-quality randomized controlled trial data in early-stage breast cancer-related lymphedema show no statistically significant limb volume reduction at 6 months compared with conservative therapy, though some patients experience subjective improvement and reduced need for compression garments. 25 In contrast, longer-term and larger cohort studies consistently report meaningful volume reductions, particularly in patients with moderate-to-severe or chronic lymphedema. 21 22 23

Given the challenges in documenting the persistent patency of clinical LVB postoperatively, the value of animal models cannot be underestimated. As noted previously, despite 100% early patency, no long-term patency was observed in an animal model of lymphedema, 11 while LVB in “normal” animals appears to achieve far better long-term patency. 10 20 An excellent systematic review of the animal model in surgical management of lymphedema documents the challenges of these essential tools to better understand the pathophysiology and potential treatments of lymphedema. 28 Unfortunately, it also demonstrates the surprising lack of quantifiable data regarding the efficacy and long-term patency of LVB in animal models of lymphedema. A more recent study using a rabbit ear lymphedema model demonstrates the feasibility of performing LVB in this situation, 29 but the lack of a control group and previous data demonstrating spontaneous reduction in lymphedema-related swelling make the interpretation of the results challenging.

## Role of Ultrahigh-Frequency Ultrasound in Planning Lymphovenous Anastomosis

Advances in imaging technology have enhanced the precision and success of LVB, with ultrahigh-frequency ultrasound (UHF-US) emerging as a valuable adjunct to traditional modalities such as ICG lymphography. Operating at frequencies above 20 MHz—typically 48 to 70 MHz—UHF-US enables real-time visualization of superficial lymphatic collectors and subdermal venules with submillimetric resolution. This imaging capability is especially useful in surgical planning, anastomotic site selection, and mapping in patients with limited or absent ICG signal.

### Visualization of Superficial Lymphatic Vessels

UHF-US allows for direct, contrast-free visualization of functioning lymphatic vessels. These typically appear as hypoechoic or anechoic tubular structures with smooth, thin walls located in the superficial subcutaneous tissue. 30 Moreover, the quality of the sonographic images correlates with histology—validating the diagnostic accuracy for selecting vessels for LVB. 31 Unlike ICG lymphography, which is limited by tissue penetration (∼1.5 cm) and requires patent lymphatic flow for contrast uptake, UHF-US can detect structurally intact collectors even in edematous, fibrotic, or postsurgical limbs where the ICG signal is compromised. 32 This has proven particularly valuable in advanced-stage lymphedema (International Society of Lymphology stage IIb–III), where ICG imaging alone may falsely suggest a lack of viable vessels. It is also notable that UHF-US, in combination with ICG lymphography, can identify the “ideal” location for conducting an LVB—where a lymphatic vessel is patent, but becomes obstructed more proximally.

### Identification of Suitable Venules

In addition to lymphatics, UHF-US allows precise mapping of subdermal venules, including measurement of diameter, wall thickness, and compressibility. With high spatial resolution, vessels as small as 0.2 to 0.3 mm in diameter can be identified and assessed for suitability as recipient veins. When combined with Doppler imaging, the absence of reflux and patency of flow can be confirmed, improving the selection of viable venules for anastomosis and reducing intraoperative uncertainty.

### Surgical Planning and Intraoperative Guidance

By simultaneously imaging both lymphatic and venous structures, UHF-US supports targeted incision placement, minimizing dissection and operative time. 33 It enhances the efficiency of multisite or multilevel LVB procedures, particularly in complex cases with altered lymphatic anatomy.

### Limitations and Future Directions

While promising, UHF-US is not without limitations. The technology is operator-dependent and requires training to accurately identify lymphatic versus other vascular structures. Scanning can be time-consuming, particularly for full-limb mapping, and device availability remains limited to specialized centers. Nonetheless, its potential to complement ICG lymphography is significant, particularly in difficult anatomical fields or cases of prior surgery. It is also worth noting that the role of conventional high-frequency ultrasound (18 MHz) in planning LVB has also been described. 34

Emerging protocols integrating UHF-US with ICG lymphography and anatomical lymphosome mapping may provide a more comprehensive framework for personalized LVB planning, reducing operative variability and improving outcomes. 35 Specifically, the combined use of ICG imaging and UHF-US provides the potential to localize the “ideal” location to perform LVB where the lymphatic remains patent (and frequently enlarged or ectatic) and provides drainage of an affected lymphosome, but becomes obstructed more proximally. The utilization of a patent, but otherwise non-functional lymphatic, provides the opportunity to improve lymphatic drainage, without disrupting a potentially functional lymphatic, which cannot increase lymphatic egress from the limb.

## The Potential of Microsurgical Robots and Exoscopes in Lymphovenous Bypass Surgery

LVB is a technically demanding supermicrosurgical procedure that requires suturing vessels often <0.8 mm in diameter. While conventional operating microscopes and manual techniques remain the standard, recent advances in **microsurgical robotics** and **exoscopes** have the potential to transform the field by improving precision, ergonomics, and accessibility.

## Microsurgical Robotics

Robotic systems specifically designed for microsurgery, such as the Symani Surgical System, provide motion scaling, tremor filtration, and enhanced precision, which are especially valuable for lymphatic anastomoses. 36 37 Early case series have demonstrated technical feasibility and safety, with acceptable patency and lymphedema outcomes. 38 Microsurgical robots may also flatten the learning curve, allowing more surgeons to adopt LVB, while providing access to anatomically challenging regions (e.g., deep lymphatics or central lymphatic reconstructions). 39 Importantly, pilot data suggest that robotic assistance may standardize outcomes across experience levels. Despite the potential advantages of these devices, there are notable limitations, including high capital costs, lack of haptic feedback, instrument size constraints, and longer operative times during the initial adoption phase. 39 40 41 Moreover, robust comparative outcome data are still lacking, with most reports limited to case series and feasibility studies. 42

## Exoscopes

High-definition 3D exoscopes (e.g., ORBEYE) offer superior ergonomics, shared operative visualization, and teaching benefits compared with conventional microscopes. 43 44 Their small footprint also frees operative space and facilitates team-based surgery. Several comparative studies suggest exoscopes are non-inferior to microscopes for supermicrosurgical anastomoses, with the added advantage of improved posture and reduced surgeon fatigue. 45 Case reports have demonstrated successful LVB using exoscopes in clinical practice. 44 Microsurgical robots and exoscopes represent promising adjuncts in the evolution of lymphatic surgery. Despite current limitations of cost, setup, and evidence base, ongoing refinements and multicenter studies will determine whether these technologies can improve the surgical management of lymphedema. As the subsequent discussion of the rheology of lymphatic flow will highlight, the ability to reduce the size of LVB may not necessarily improve the physiological treatment of lymphedema.

## Future Directions and Unanswered Questions

From the early conception of the potential to address secondary lymphedema by “bypassing” the obstruction with distal redirection to the venous system to the current widespread adoption of LVB in the management of lymphedema, several essential developments have occurred ( Table 1 ). First, the anatomy of the lymphatic system, including the topographical organization of lymphosomes, provides the basis for understanding the anticipated impact of proximal lymphatic disruption, as well as designing potential reconstructive interventions. Second, early surgical innovators, such as Isao Koshima, demonstrated the technical feasibility of performing LVB. Finally, there are both objective and PROMs that suggest that LVB surgery can improve the clinical state of individuals with lymphedema. Despite these landmark developments in the surgical management of lymphedema, a large number of questions regarding the “ideal” role of this surgery persist. The understanding of patient-specific lymphatic anatomy and the response to lymphatic injury, the technical and rheological principles of LVB, and the specific outcome metrics to understand the development, progression, and treatment of lymphedema will be explored.

**Table 1:** Evolution of lymphovenous bypass: Historical foundations, current practice, and future directions

Domain	Historical foundations	Current practice	Future directions
Conceptual basis	Early experimental attempts to divert lymph into the venous system (1950s–1970s)	Established physiologic principle for secondary lymphedema management	Integration of lymphatic biology with personalized surgical planning
Anatomical understanding	Limited appreciation of lymphatic territories and lymphosomes	Use of ICG lymphography and UHF-US to identify functional, patent lymphatics	Multimodal 3D lymphatic mapping for individualized operative strategy
Technical approach	Early macroscopic lymphatic–venous shunts with poor durability	Supermicrosurgical LVB with vessels ≤0.8 mm; refined anastomotic techniques	Robotic supermicrosurgery, enhanced visualization, and automated suturing systems
Model systems	Animal studies demonstrating transient patency but early occlusion	Validated high-fidelity training models and in vivo perfused simulations	Dynamic computational and perfusion-based models for physiologic validation
Patient selection	Empirical, often late-stage lymphedema with limited success	Stage-specific and anatomy-driven selection using functional imaging	Predictive modeling based on lymphatic morphology and regenerative capacity
Outcome assessment	Qualitative assessment of swelling reduction	Combined objective (volume, imaging) and subjective (PROMs) outcomes	Standardized, multidimensional outcome metrics integrating imaging and QoL data
Preventive surgery	Not attempted; reactive management only	Immediate lymphatic reconstruction (ILR) at lymphadenectomy is gaining adoption	Selective ILR guided by risk stratification and real-time lymphatic mapping
Scientific basis	Limited understanding of lymphangiogenesis or flow dynamics	Recognition of rheological principles (pressure, radius, flow resistance)	Molecular modulation of lymphangiogenesis and scaffold-based vessel regeneration

Abbreviations: ICG, indocyanine green; LVB, lymphovenous bypass; PROM, patient-reported outcome; QoL, quality of life; UHF-US, ultrahigh-frequency ultrasound; 3D, three-dimensional.

## Response to Lymphatic Injury and Patient-Specific Lymphatic Anatomy

Lymphangiogenesis—the formation of new lymphatic vessels—occurs after lymphadenectomy as part of the tissue's attempt to restore lymphatic drainage. Animal and human studies demonstrate that the degree and pattern of lymphangiogenesis, as well as the ability to form functional collateral pathways, are highly variable and are strongly influenced by preexisting anatomical variations in the lymphatic system. 46 47 48 49 50 Experimental models show that after lymphadenectomy, there is an initial period of impaired lymphatic contractility and drainage, followed by partial restoration of function through both regeneration of lymphatic vessels and co-opting of collateral pathways. The speed and completeness of this process depend on the local microenvironment, the presence of viable lymphatic endothelial cells, and the ability to upregulate molecular drivers of lymphangiogenesis such as VEGF-C/VEGFR-3 signaling. 46 51 In some cases, robust lymphangiogenesis and collateralization can restore near-normal drainage and reduce the risk or severity of lymphedema, while in others, inadequate or aberrant vessel formation leads to persistent dermal backflow and chronic lymphedema. 47 48 52 This phenomenon of lymphangiogenesis, in conjunction with more extensive collateral pathways, is a major obstacle in developing suitable animal models of lymphedema. 28 Anatomical variations—such as a sparse baseline lymphatic network, limited collateralization, or segmental/proximal flow restriction—are associated with poor lymphangiogenic response and higher risk of severe, persistent lymphedema after lymphadenectomy. 48 52 These same factors predict suboptimal outcomes after physiologic surgical interventions like LVA, as successful bypass requires the presence of functional, regenerating lymphatic channels. 52 In summary, the capacity for lymphangiogenesis and the anatomical pattern of lymphatic regeneration after lymphadenectomy are critical determinants of lymphedema risk and the success of physiologic surgical interventions, with poor baseline lymphatic anatomy and limited collateralization predicting worse outcomes. 46 47 48 49 52

The critical role of collateral pathways and lymphangiogenesis has been well-described by Suami. 48 These insights provide a framework for understanding possible explanations for why only a portion of individuals who undergo lymphadenectomy develop lymphedema. This provides potential opportunities to increase the probability of successful lymphangiogenesis, such as by decreasing scarring within the zone of lymphatic disruption. It may also provide the opportunity to image patients preoperatively, with ICG lymphography, to try and determine who may be at greater risk of developing lymphedema. One specific example of patient-specific anatomy and the risk of developing lymphedema is the relationship of the lateral upper arm lymphatic channels as defined by the presence, continuity, and connectivity of these channels to the forearm and alternative drainage pathways. Patients with a short bundle or absent lateral upper arm lymphatic channel—meaning the channel is present but not contiguous with the forearm, or entirely absent—are at increased risk of developing lymphedema after axillary lymphadenectomy. This is because the lateral upper arm channel serves as an accessory pathway that can bypass the axilla and drain to the deltopectoral or supraclavicular nodes, providing a protective route for lymphatic drainage if the axillary pathway is disrupted. 53 54 55 When this channel is poorly developed or lacks connection to the forearm, the arm is more reliant on the medial upper arm pathway, which is more likely to be disrupted during axillary surgery. In the context of anatomical lymphatic variations, such as non-linear or sparse lymphatic networks, the risk is further increased, as these patients have less capacity for collateralization and alternative drainage. 53 54 Impaired lymphangiogenesis and poor lymphatic regeneration after lymphadenectomy compound this risk, as the absence of robust preexisting pathways limits the ability to restore effective lymphatic flow, leading to persistent dermal backflow and chronic lymphedema. 48 49 50

Despite the independent importance of lymphangiogenesis and patient-specific lymphatic anatomical differences in the risk of developing clinical lymphedema, from the perspective of management with LVB, it highlights two important considerations. First is immediate lymphatic reconstruction (LVB at the time of lymphadenectomy), a potential opportunity to prevent the secondary development of lymphedema? The evidence for this intervention will be reviewed in the subsequent section, but it is already apparent that, given the rate of lymphedema following lymphadenectomy (∼15–25%), the routine usage of immediate lymphatic reconstruction (ILR) will necessitate performing this procedure in 75% to 85% of patients who would not have required the procedure. The second important consideration relates to the anatomy of the remaining lymphatics in individuals with lymphedema, and how they should be selected for potential bypass. A standard recommendation would typically be “Specific lymphatic vessels should be selected for bypass procedures in individuals with lymphedema based on the identification of functional, patent lymphatic channels that are not sclerosed, with preference for those that provide the most direct drainage from the affected limb and have the greatest likelihood of successful anastomosis.” Although this description of planning for LVB appears intuitively correct, it is questionable to disrupt a “functional” lymphatic in an individual who is achieving lymphatic egress from their extremity with an altered lymphatic drainage, and is likely highly reliant on these remaining functional lymphatics. Ideally, functional lymphatics would be selectively preserved, and non-functional but patent lymphatic channels would be utilized for LVB procedures, thereby increasing the potential drainage from the extremity. This is a conceptual shift from the earlier execution of LVB, and relies on the long and progressive discoveries of the pioneers in the field, as well as the advanced imaging (UHF-US and ICG) available to surgeons to attempt this more anatomically directed application of LVB.

## The Rheological and Technical Considerations of Lymphovenous Bypass

The Hagen–Poiseuille equation ( *Q* = πΔ *Pr* ^4^ /8 *ηL* ) describes how fluid flow ( *Q* ) through a cylindrical vessel is determined by the pressure gradient (Δ *P* ), vessel radius ( *r* ), fluid viscosity ( *η* ), and vessel length ( *L* ). In LVB surgery for lymphedema, this relationship is directly relevant to surgical outcomes. Lymphatic fluid viscosity is often increased in lymphedema due to higher protein content, which reduces flow for a given pressure gradient and vessel diameter. Flow dynamics are further impacted by the degree of lymphosclerosis: Sclerosed lymphatics have reduced diameter and increased wall stiffness, both of which decrease flow exponentially, as flow is proportional to the fourth power of the radius. Therefore, selecting less sclerotic, larger-diameter lymphatics is critical for maximizing flow through the anastomosis and achieving successful antegrade lymph-to-vein drainage. 56 57

Pressure gradients must favor lymphatic-to-venous flow. A larger diameter difference between the lymphatic and the recipient vein, with the lymphatic being larger and less sclerotic, and the vein being smaller and low pressure, optimizes the pressure gradient and reduces the risk of venous reflux. The Venturi effect, as described in recent studies, can further enhance initial drainage by creating a localized low-pressure zone at the anastomosis. 56 58 For example, the flow across a 0.5-mm anastomosis is approximately 2.5 times greater than the flow across a 0.4-mm anastomosis. Clinical data confirm that anastomoses using lymphatic vessels ≥0.5 mm in diameter are associated with significantly better postoperative volume reduction and functional outcomes, and that more sclerotic or smaller lymphatics are associated with higher rates of venous reflux and anastomotic failure. 56 57 In summary, the Hagen–Poiseuille equation underscores the importance of selecting lymphatics with the largest possible functional diameter, minimal sclerosis, and high flow velocity, and matching them to small, low-pressure veins to maximize flow and surgical success in LVB for lymphedema. 56 58 59 Although the goal of utilizing the largest possible lymphatic vessels for bypass is readily understandable, this is not always clinically practical. Nevertheless, the use of UHF-US in conjunction with ICG lymphography can aid the surgeon in trying to determine locations where this goal can potentially be achieved.

As noted previously, the previous basic science animal studies demonstrated that despite early patency, several LVBs failed over time. 10 11 This appeared to influence the subsequent clinical application and development of LVB for individuals with lymphedema. This resulted in the approach of multiple anastomoses to try and mitigate against the tendency for anastomotic failure over time. Unfortunately, the goal of multiple anastomoses did not necessarily maximize the potential volume of egress, as many of the LVB were well below the 0.5-mm size, but also did not permit the anatomic specificity that focused on lymphosomal regions of disruption. Although there have been previous anatomic recommendations about where LVB may be more successful, such as the superior edge knee incision point 60 for lower extremity lymphedema, the future of LVB likely rests in patient-specific examination of the involved extremity to determine the “ideal” locations for these procedures. Although each patient will present a unique pattern of dysfunction and lymphangiogenesis, the preferred locations of LVB would address the specific lymphosomes that are obstructed, would avoid the disruption of functional lymphatics, and use the largest patent, non-sclerotic lymphatics for bypass. Achieving all of these goals is difficult and may not be achievable in most situations, but our imaging tools make these goals far more attainable than they were when the pioneers of this field demonstrated the feasibility of LVB.

One example of a technical advancement that likely comes remarkably close to providing a particularly advantageous intervention for specific patients with lower extremity lymphedema is the modern conception of the lymph node to vein bypass. 61 In patients who have undergone pelvic lymphadenectomy, the remaining inguinal lymph nodes can provide an ideal location to redirect the lymphatic fluid draining from the lower extremities with a technically feasible procedure utilizing larger veins that can potentially address the drainage from multiple lymphosomes without disrupting functioning lymphatics. There are specific requirements to ensure the viability of the procedure, but it holds great potential to improve lymphedema within this specific patient population. This provides one example of an opportunity to explore specific approaches to LVB to address lymphedema, utilizing the developing understanding of this condition in conjunction with the expanding tools to better evaluate a given patient's pathology to determine the most beneficial intervention.

## Immediate Lymphatic Reconstruction—Can We Prevent the Development of Lymphedema?

Despite the dramatic technical advancements in the area of LVB, it remains clear that we cannot consistently “cure” lymphedema. Accordingly, several surgeons have suggested that the greatest potential benefit of LVB is to prevent the development of lymphedema by using these techniques at the time of lymphadenectomy and performing ILR. 62 63 64 65 66 67 68 This technique is also referred to as Lymphatic Microsurgical Preventive Healing Approach (LYMPHA).

Recent systematic reviews and meta-analyses pooling prospective and retrospective cohorts suggest ILR is associated with markedly lower odds of lymphedema overall, with potential attenuation over longer follow-up and evidence of publication bias; these syntheses underscore the need for standardized protocols and extended surveillance. 67 68 69 Despite these generally positive results, more recent well-designed studies are less clear regarding the long-term benefit of ILR. In a matched cohort with a median follow-up of ≥4 years (57 vs. 63 months), the cumulative lymphedema incidence was similar with versus without LYMPHA: 31.1% versus 33.3% ( *p* > 0.99), suggesting loss of preventive effect over time; no benefit was seen in obesity or postradiation subgroups. 70 Similarly, a pragmatic two-site prospective cohort (surgeon-level assignment; intention-to-treat 131 axillary lymph node dissection (ALND) + ILR vs. 99 ALND): Using multiple ascertainment strategies (objective limb volume thresholds, self-report, provider documentation, ICD-10), there was no statistically significant difference in lymphedema rates between ALND with ILR and ALND alone after multivariable/propensity adjustment. 71 Finally, one of the most comprehensive and well-designed prospective studies to address this question 63 that demonstrated a significant benefit of ILR at 12 months, failed to observe this ongoing benefit at 24 months (ASRM 2025 meeting). Taken as a whole, it is unclear if ILR will realize the goal of preventing secondary lymphedema. In some regard, this is not dissimilar to the early animal results that demonstrated 100% patency of LVB in a similar canine model at 7 days, but occlusion of all anastomoses by 21 days. 11 It is also worth reiterating that despite lymphadenectomy and frequently receiving adjuvant radiotherapy treatment, many patients do not develop lymphedema. There appears to be a large range of incidence of secondary lymphedema following lymphadenectomy, which is likely within the 10% to 20% range at 1 year, increasing up to 20% to 30% within 5 years. Although there are multiple factors that appear to influence these outcomes, such as the impact of radiotherapy and the greater incidence of lymphedema following removal of a larger number of nodes, it is apparent that the deployment of ILR to all patients will result in 50% or more undergoing surgery, unlikely to provide added benefit, but does utilize valuable health care resources.

## Outcomes Following Lymphovenous Bypass—What is a Good Result?

Although the desired physiological outcome following LVB is clear—to restore lymphatic drainage by creating direct connections (anastomoses) between obstructed lymphatic vessels and adjacent venules, thereby diverting lymphatic fluid into the venous circulation, the method or tools to determine the outcome of these interventions is far less clear. Should we examine more objective outcomes, such as volumetric changes or improvements in transport times on lymphoscintigraphy, or should we focus on PROMs, such as those obtained using validated tools such as the LYMPH-Q Upper Extremity for upper limb lymphedema or the LYMQOL (Lymph Quality of Life Measure for Limb Lymphedema)? Alternatively, we could utilize more simplistic binary outcomes, such as the need for compression, as this presents both functional and psychological challenges for individuals experiencing lymphedema. This is not simply a theoretical exercise, as several studies have reported positive outcomes of LVB with regard to PROMs, 26 27 in the absence of evidence of concurrent volume reductions. This creates an important question regarding the disparities observed between objective measures of improved lymphatic drainage (reduced limb volume or improved transport index) and subjective measures or PROMs. Two potential explanations for the divergence of these measures are that our volumetric measurements are of inadequate sensitivity or alternatively, that we are observing a placebo effect with regard to the PROMs. The magnitude of placebo effects in PROMs is generally small to moderate, but varies substantially by condition and outcome type. In high-quality randomized trials across a broad range of clinical conditions, pooled standardized mean differences (SMDs) for PROMs typically range from −0.26 to −0.39, indicating a small but statistically significant improvement attributable to placebo interventions, with effects being larger for patient-reported than observer-reported outcomes. 72 73 74 Importantly, placebo effects are consistently larger for PROMs than for objective or observer-reported outcomes (SMD for observer-reported outcomes ≈ −0.13 to 0.09). 72 73 74 The magnitude of placebo effects is influenced by factors such as trial design, patient expectations, and the nature of the outcome measure. This is not to indicate that PROMs should not be utilized, but the well-established risk of the placebo effect on this component of data in this patient population highlights the importance of convergent data (both objective and subjective) to ensure we are observing postsurgical changes that truly reflect a clinically meaningful physiological change.

The importance of obtaining objective outcome measures is also clear when we observe the typically modest effect size of LVB (or LVA) in terms of objective limb volume reduction, with mean reductions in excess limb volume generally ranging from 6% to 33% at 1 year, depending on patient selection, disease stage, and measurement method. 5 75 76 Meta-analyses and large cohort studies report pooled mean reductions in excess limb volume of approximately 32.7%, but individual studies often show lower average reductions, especially in chronic or advanced-stage lymphedema. 76 For upper extremity lymphedema, mean volume differential reductions after LVB are reported as 6.2% ± 5.8% at 1 year in controlled trials, while some prospective series report reductions of 33% to 42% at 3 to 12 months, with greater effect in early-stage disease. 5 76 Lower extremity lymphedema tends to show smaller reductions, typically 14% to 22% at 6 to 12 months. 77 78 79 Importantly, several randomized and prospective studies demonstrate that PROMs (quality of life, symptom burden, compression garment use, and cellulitis frequency) often improve significantly even when objective limb volume reduction is minimal or not statistically significant. 25 26 27 For example, in a randomized trial, LVB led to significant improvements in physical and mental function domains, but no significant change in limb volume at 6 months. 25 Similarly, large cohorts show improved quality of life and reduced compression garment use without significant change in limb circumference. 26 27 In summary, the typical effect size for limb volume reduction after LVB is modest (6–33%), and improvements in PROMs may occur independently of measurable volume reduction. 5 25 26 27 75 76

Taken as a whole, the field of physiological lymphatic reconstruction has developed exponentially over the past number of decades, with pioneers demonstrating the potential for the consistent performance of LVB. In the near future, we should be better able to ascertain those patients who will experience the greatest potential benefit from this surgery, and also identify those patients that might be better served with other physiological and non-physiological surgery, or other interventions. It is also worth noting the invaluable impact of technological advances, such as improved microsurgical instruments, microscopes, ICG imaging, and UHF-US, that permitted the rapid development of LVB surgery. It is distinctly possible that the recent development of microsurgical robots and further growth of enhanced visualization will further propel the technical aspects of this procedure. Nevertheless, the conceptual and evidence-directed aspects of LVB will be essential to direct when and how we consider this intervention for specific patients.

## Narrative Review Methodology

This manuscript undertook a narrative review of the available literature on LVB using an extensive search strategy primarily utilizing PubMed and Google Scholar. The literature was then analyzed for its impact and volume of citation to help direct the inclusion in the final manuscript. Additional separate literature reviews were required to provide relevant publications on the topics of microsurgical robots, exoscopes, lymphangiogenesis, and PROMs.

## Footnotes

* Some surgeons have now shifted to an approach utilizing one or two anastomoses with highly detailed anatomic guidance.
